# Transition readiness in adolescents with congenital heart disease during transfer to adult care: A secondary analysis of the STEPSTONES randomized controlled trial

**DOI:** 10.1016/j.hctj.2026.100154

**Published:** 2026-10-13

**Authors:** Hilda Malmesjö, Mariela Acuna Mora, Ewa-Lena Bratt, Carina Sparud-Lundin, Philip Moons, Markus Saarijärvi

**Affiliations:** a Karolinska Institutet, Department of Neurobiology Care Sciences and Society, Huddinge, Stockholm, Sweden; b University College of Borås, Faculty of Caring Science Work Life and Social Welfare, Västra Götaland, Borås, Sweden; c University of Gothenburg, Institute of Health and Care Sciences, Västra Götaland, Gothenburg, Sweden; d Children's Heart Center, Sahlgrenska University Hospital, Västra Götaland, Gothenburg, Sweden; e University of Cape Town, Department of Paediatrics and Child Health, Cape Town, South Africa; f KU Leuven, Department of Public Health and Primary Care, Leuven, Flanders, Belgium; g Department of Cardiology, Danderyd University Hospital, Stockholm, Stockholm, Sweden

**Keywords:** Adolescents, Congenital, Heart disease, Empowerment, Randomized controlled trial, Transfer, Transition

## Abstract

**Purpose:**

Adolescents with congenital heart disease (CHD) are at risk of discontinuity of care during transfer from pediatric to adult services. Transition readiness is considered essential for a safe transfer. This study evaluated the effect of the STEPSTONES person-centered transition program on adolescents’ self-reported transition readiness compared with usual care.

**Design and methods:**

This secondary analysis was based on a randomized controlled trial conducted at two Swedish CHD centers. A total of 139 adolescents (age 16 years) were randomized to intervention (n = 70) or usual care (n = 69). The intervention group received the STEPSTONES program in addition to usual care over two years. Transition readiness was assessed at ages 16 (T0), 17 (T1), and 18.5 (T2) using the Readiness for Transition Questionnaire. Between-group differences were analyzed with Mann–Whitney U tests, and longitudinal changes were examined using mixed-design ANOVA.

**Results:**

At T2, the intervention group demonstrated significantly higher overall transition readiness compared with controls (p = .011–.015), with a small effect size (r ≈ .23–.24). A significant main effect of time was found (p < .001), indicating increased readiness across adolescence in both groups. No significant group-by-time interaction was detected (p = .177), and no differences were identified at the level of individual questionnaire items.

**Conclusions:**

The STEPSTONES program was associated with higher overall transition readiness at the time of transfer, although effect sizes were small. Structured, person-centered transition programs may enhance preparedness for adult care.

**Practice implications:**

Pediatric nurses are key in supporting transition readiness. Structured transition programs may strengthen adolescents’ overall preparedness for adult-oriented healthcare.

**Trial registration:**

ClinicalTrials.gov NCT02675361.

## Highlights

• STEPSTONES increased overall transition readiness for adolescents with CHD.
• Readiness improved over time in both study groups.
• No significant effects were found at item level.
• Findings support structured transition programs in pediatric cardiology.

## Introduction

Congenital heart disease (CHD) affects approximately 9 per 1000 live births globally, resulting in approximately 4.18 million children under the age of five with the condition.[1], [2] CHD includes structural abnormalities of the heart or intrathoracic great vessels of functional significance.^3^ Although disease severity varies, most individuals require medical or surgical treatment and lifelong follow-up.^4^ Due to advances in pediatric cardiology and cardiac surgery, more than 97% of children with CHD in high-income countries now survive into adulthood.^5^ Consequently, the adult population is now surpassing the pediatric population.^6^ Lifelong specialized surveillance is required because of the risk of late complications, with follow-up intensity determined by disease complexity.^4^ In addition to medical monitoring, adolescents and young adults often need support related to lifestyle, mental health, education, and reproductive health, areas in which pediatric nurses play a central role.[4], [7]

As survival improves, increasing numbers of adolescents with CHD must transfer from pediatric to adult healthcare. Transition is defined as the purposeful process of preparing adolescents with chronic conditions to assume responsibility for their health in adulthood.^8^ This period coincides with significant developmental changes. Ongoing maturation of executive functions heightened emotional reactivity, and evolving autonomy may challenge adolescents’ capacity for self-management and adherence.^9^ Young people with chronic illness also strive to maintain a sense of normality, which can influence engagement in care.^10^

Transfer to adult care typically occurs at 18 years of age, regardless of individual readiness.^4^ Pediatric services are often family-centered, whereas adult care emphasizes autonomy. Adolescents frequently report feeling insufficiently prepared, inadequately informed, or excluded from transition planning.[11], [12], [13] Globally, 26.1% of young people with CHD experience interruptions in follow-up during transition. Discontinuity of care is associated with adverse outcomes; young adults who lapse in follow-up are 3.1 times more likely to require urgent cardiac intervention.^7^

International guidelines emphasize structured, coordinated, and person-centred transition processes.[4], [7], [14] To date, several randomized controlled trials (RCT) and quasi-experimental studies across various chronic conditions have demonstrated the effectiveness of transition programs in improving a range of PROM and PREM outcomes.[15], [16], [17], [18] The STEPSTONES-CHD-project (Swedish Transition Effects Project Supporting Teenagers with chrONic mEdical conditionS–Congenital Heart Disease) aimed to contribute to this body of knowledge by evaluating a person-centered transition program designed to strengthen adolescents’ preparedness for adult care.^19^ The RCT demonstrated that STEPSTONES improved patient empowerment.^20^ As empowerment is closely linked to transition readiness,^21^ defined as adolescents’ preparedness to assume responsibility for health, healthcare and transfer to adult services, understanding how such programs influence readiness is clinically important.^22^ Pediatric nurses are central to promoting self-management, autonomy, and continuity of care; therefore, identifying which domains of transition readiness are affected by structured interventions is highly relevant to nursing practice. The aim of this study was to evaluate the efficacy of the STEPSTONES transition program for adolescents aged 16–18.5 years with congenital heart disease (CHD), compared with usual care, in improving their self-reported readiness to transition to adult healthcare. It was hypothesized that adolescents participating in the two-year transition program would demonstrate higher transition readiness scores than adolescents receiving usual care.

## Materials and methods

### Study design

A hybrid experimental design combining an RCT within a longitudinal observational study was conducted, resulting in a three-arm design (Fig. 1).^19^ The study was performed at seven CHD centers in Sweden. At two centers, adolescents were randomized to an intervention group (IG) or control group (CG). The remaining five centers served as a contamination control group (CCCG), where no intervention was introduced to minimize the risk of contamination bias.^19^

**Fig. 1:**
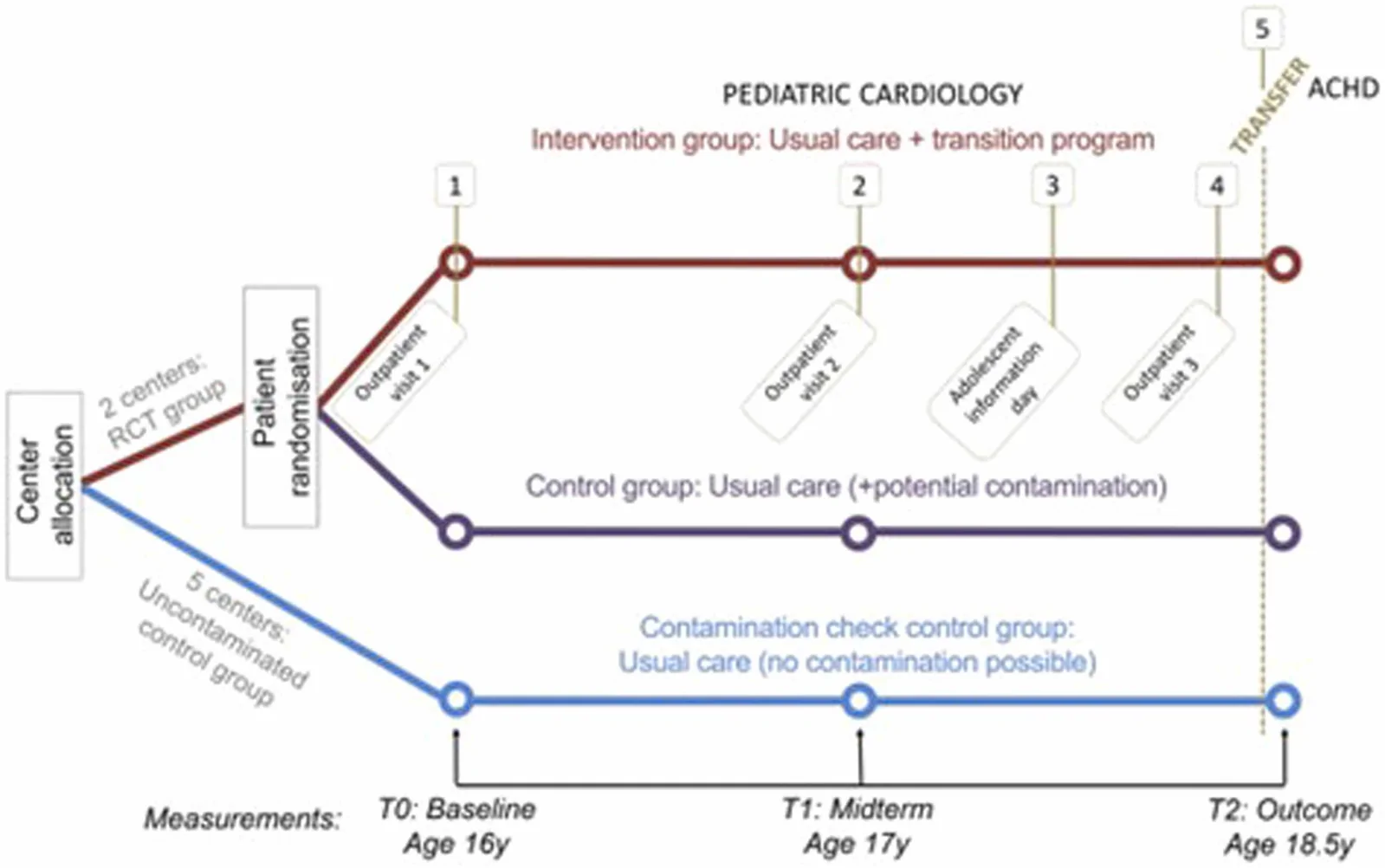
STEPSTONES-CHD study design.

Previous analyses demonstrated no differences in outcomes between the CG and CCCG, indicating no contamination effect.^20^ Therefore, only data from the two RCT groups were included in the present analyses. Reporting of the study followed the CONSORT guidelines.^23^

## Sample size

Sample size calculation was based on the primary outcome (patient empowerment), with an expected improvement of 5.25 points (half a standard deviation) on a 15–75 scale.^24^ With α = 0.05 and 80% power, 63 participants per group were required. Allowing for 10% attrition, 70 participants per group were targeted.^19^ As transition readiness was a secondary outcome, power was not calculated for this measure; effect sizes were therefore estimated post hoc using Cohen’s d.

## Participants and recruitment

Eligible participants were 16-year-old adolescents with mild, moderate, or severe CHD,^25^ defined as “a gross structural abnormality of the heart or the intrathoracic great vessels that actually or potentially [is] of functional significance”.^3^ Adolescents were required to be Swedish-speaking, literate (i.e., being able to read, comprehend and speak Swedish), and scheduled for follow-up at an Adult Congenital Heart Disease (ACHD) clinic. Eligible participants were identified through the Swedish Registry of Congenital Heart Disease (SWEDCON).^26^

Exclusion criteria included cognitive impairment that prevented the adolescent from answering the questionnaire independently, acquired or non-structural heart conditions, or previous heart transplantation. Parents were invited to participate, but adolescent participation was not contingent on parental participation. Recruitment was conducted by a transition coordinator (TC) or data collection officer (DCO). Participants received a cinema ticket at each data collection point.^19^

## Randomization

Participants at the two RCT centers were randomized 1:1 to IG or CG using a web-based system with stratified block randomization and randomly varying block sizes. Stratification was performed by study center and disease complexity to ensure balanced allocation and prevent predictability.^20^

## 1 Intervention

### STEPSTONES transition program (IG)

The STEPSTONES program was developed using intervention mapping, a structured approach to health program development.[27], [28] The program was adapted from a Belgian transition intervention for adolescents with juvenile idiopathic arthritis^29^ and tailored to the Swedish context.

The intervention comprised eight components delivered across five structured steps (Fig. 2). Components included^1^: transition coordinator^2^; written person-centered transition plan^3^; person-centered information and education^4^; availability via telephone and email^5^; introduction to adult services^6^; parental guidance^7^; peer support; and^8^ structured transfer to adult care.^19^

**Fig. 2:**
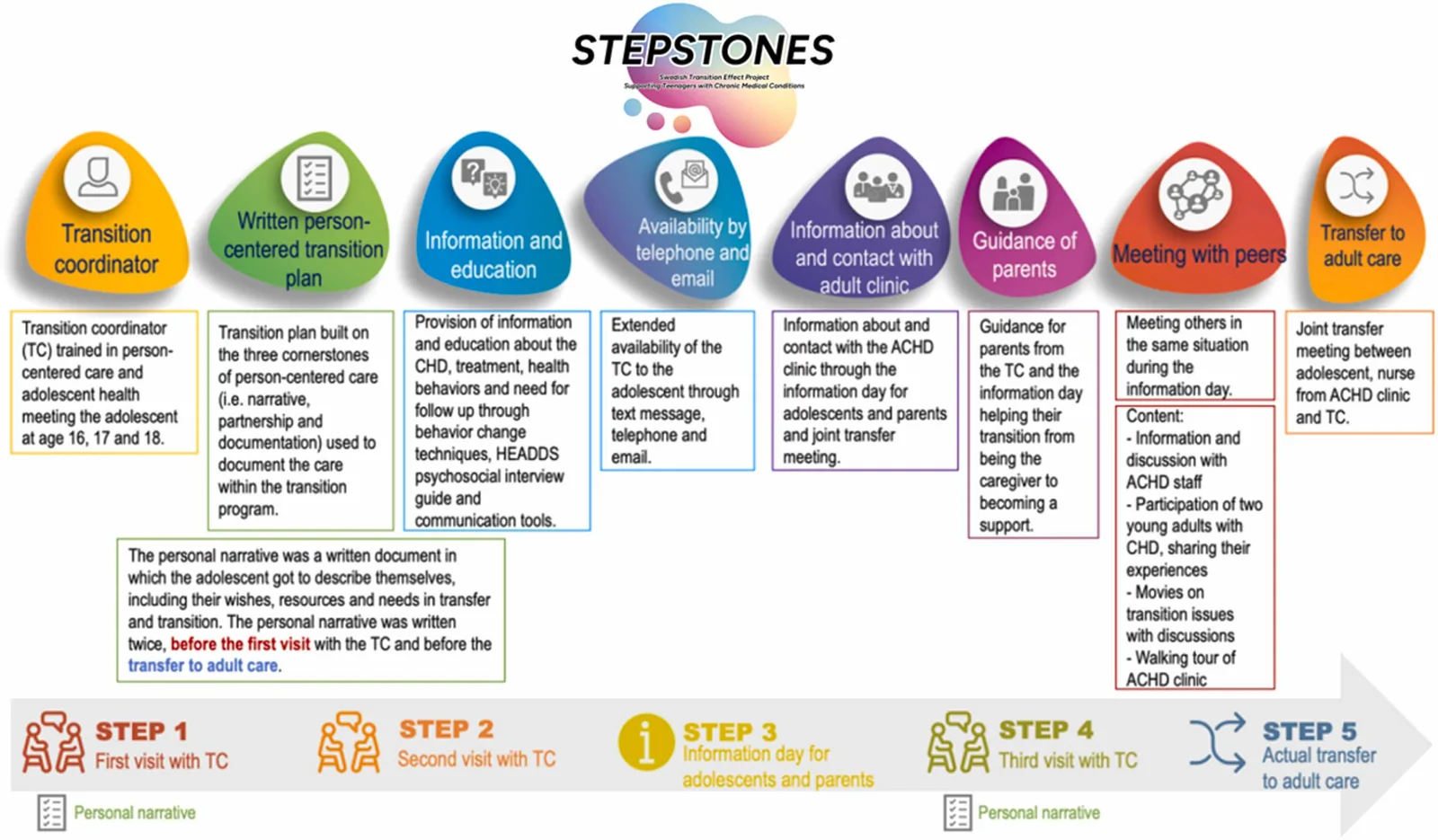
STEPSTONES transition program.

In practice, adolescents met with the TC at three individual visits, participated in an information day with parents, and completed a structured transfer process. TCs were experienced pediatric nurses who completed a four-day training program before delivering the intervention.^19^

## Usual Care (IG, CG

All participants received standard care, consisting of regular outpatient follow-up with a pediatric cardiologist and nurse. Visit frequency was determined by disease complexity.^25^ No participating center had a formal transition program during the study period. In Sweden, the healthcare system is publicly funded, and CHD care is organized and primarily delivered at university hospitals. Transfer to adult care occurs at age 18.

## Blinding

Blinding was not feasible due to the nature of the intervention. To minimize contamination, TCs were not involved in the care of CG participants, and staff not delivering the intervention were not informed of its components. TCs and DCOs involved in implementation and data collection did not participate in study design or statistical analyses.^20^

## Data collection

Participants were followed over 2.5 years at ages 16 (T0), 17 (T1), and 18.5 (T2). Adolescents completed the Readiness for Transition Questionnaire (RTQ) at each time point.^22^ Additional measures (empowerment, health behaviors, CHD knowledge, quality of life, and patient-reported health) were collected but are not included in the present analysis.^19^

IG participants completed questionnaires prior to clinic visits or on-site. CG participants received questionnaires by post. Up to four reminders were sent according to the Dillman method to reduce attrition.^19^

## Outcome measure

Sociodemographic data (Table 1) was collected at baseline. Clinical data (i.e., CHD diagnosis, Table 1) were retrieved from medical records.^20^

**Table 1:** Demographic and clinical characteristics of participating adolescents.

	***n*****= (%)**	**IG, n = 70**	**CG, n = 69**	***p***
**Female**	60 (45.1)	29 (41.4)	32 (46.4)	0.761
**Complexity of the CHD**				0.358
Mild	15 (11.3)	10 (14.3)	7 (10.1)	
Moderate	83 (62.5)	38 (54.3)	47 (68.1)	
Severe	35 (26.3)	22 (31.4)	15 (21.7)	
**Taking medication due to CHD**	29 (21.8)	16 (22.9)	13 (18.8)	0.412
**Other congenital disorders**	25 (18.8)	10 (14.3)	15 (21.7)	0.418
**Born in Sweden**	117^88^	55 (78.6)	62 (89.9)	0.473
**Education**				0.101
Elementary school	95 (71.4)	51 (72.9)	44 (63.8)	
Secondary school	34 (25.6)	13 (18.6)	21 (30.4)	
Other	1 (0.8)		1 (1.4)	
Missing	3 (2.3)	6 (8.6)	3 (4.3)	
**Living condition**				0.521
Living with both parents	95 (71.4)	42^60^	53 (76.8)	
Living alternately with mother or father	17 (12.8)	9 (12.9)	8 (11.6)	
Mother	13 (9.8)	7^10^	6 (8.7)	
Father	4 (3.0)	3 (4.3)		
Missing	4 (3.0) ??	9 (12.9)	1 (1.4)	

## Readiness for transition questionnaire (RTQ)

Transition readiness was defined as the adolescent’s preparedness to assume responsibility for healthcare and transfer to adult services. The RTQ includes adolescent and parent versions and consists of two sections. The first Section (2 items) assesses overall readiness using a four-point scale (total score 2–8). The second Section (10 items) evaluates responsibility for healthcare tasks and parental involvement using five-point Likert scales (total score 10–40). Higher scores indicate greater adolescent responsibility and independence.^22^

The RTQ demonstrated good internal consistency (Cronbach’s α 0.79–0.94) and convergent validity in adolescents with kidney transplantation.^22^ The Swedish version was translated using forward–backward procedures, with cultural adaptation including removal of the insurance item as this was not deemed appropriate for the Swedish context and addition of one responsibility item.^30^

## Statistical analysis

Analyses were conducted using IBM SPSS Statistics version 31.0. Data were analyzed according to intention-to-treat (ITT) and full analysis set (FAS) principles. To be part of the FAS, participants had to have valid data at baseline and end point. Multiple imputation was used to handle missing data in the ITT. Categorical variables were presented as frequencies and percentages. Continuous variables were reported as median and interquartile range (IQR) and mean rank. Between-group differences were analyzed using chi-square tests and Mann–Whitney U tests. Bonferroni correction was applied for multiple item analyses. Changes over time were examined using mixed-design ANOVA to assess within-group effects, between-group effects, and interaction effects. Sphericity was met (χ²^2^ = 2.57, p = 0.276). Effect sizes were interpreted using Cohen’s d (0.2 small, 0.5 moderate, 0.8 large) and partial eta squared (η²p 0.01 small, 0.06 moderate, 0.14 large). Statistical significance was set at p < 0.05.

## Ethical considerations

The study followed the Declaration of Helsinki^31^ and was approved by the Regional Ethical Review Board in Gothenburg, Sweden (No. 931–15, T-417–16, T-435–17). Written informed consent was obtained from adolescents and parents prior to participation. Participants could withdraw at any time. Although 16-year-olds are legally able to consent under Swedish law, ethical considerations regarding voluntariness and comprehension were carefully addressed, whereby TCs performed a screening of eligibility by reading the patients electronic medical records and by telephone. The intervention posed minimal risk, consisting of structured nurse-led support without physical procedures. TCs referred participants to appropriate professionals when needed.^32^ Registry data from SWEDCON were used in accordance with opt-out procedures. Confidentiality was ensured through coded data storage and secure handling procedures.^20^

## Results

### Participant flow

Of 357 eligible adolescents, 218 were excluded, and 139 were randomized (70 to the IG and 69 to the CG, Fig. 3). During follow-up, 16 participants withdrew from the IG and 8 from the CG. Participant attrition has been reported in a previous publication.^10^

**Fig. 3:**
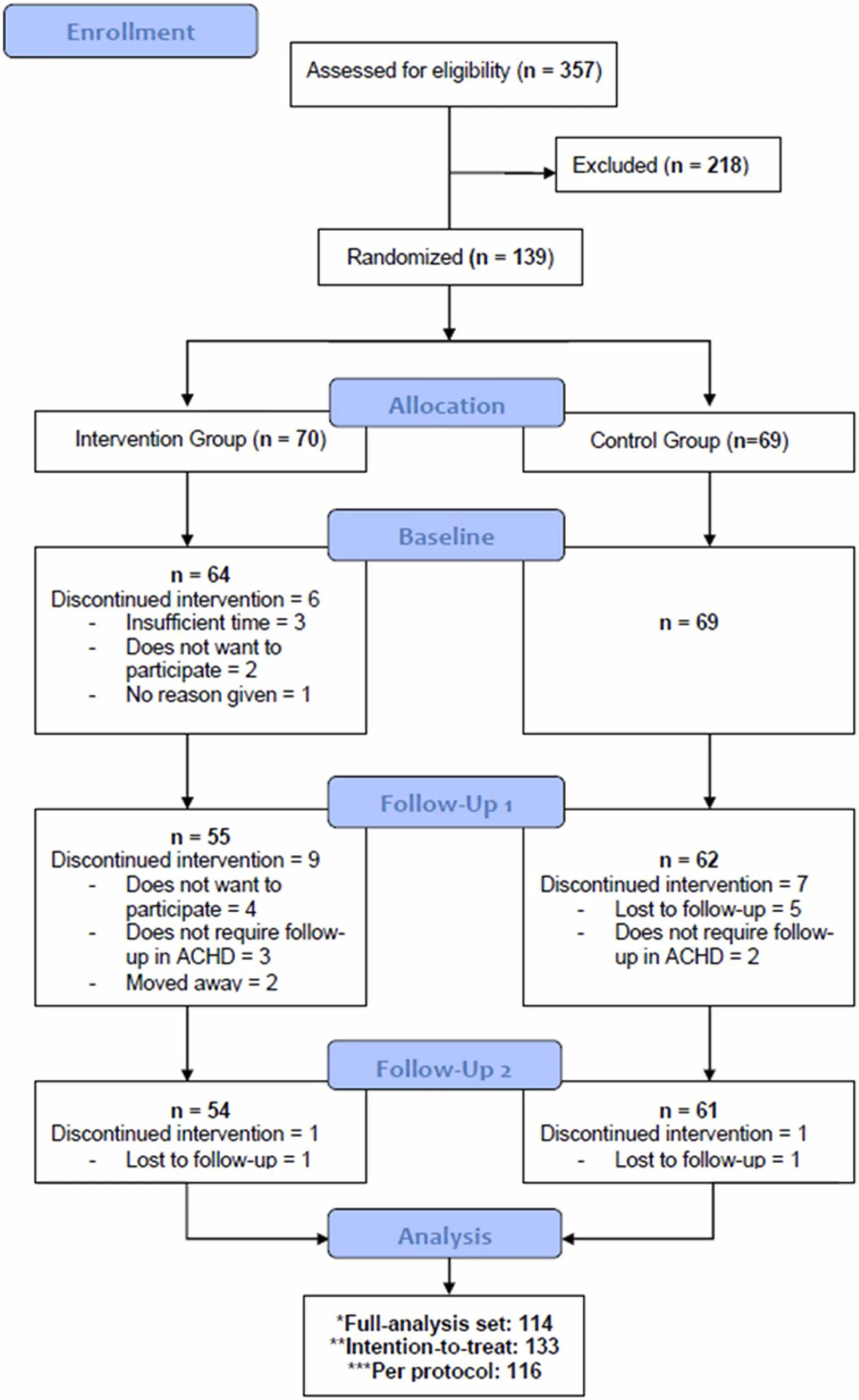
Flow chart of participant inclusion and analysis. *Full-analysis set (FAS): participants with baseline and T2 data. Intention-to-treat (ITT): all randomized participants with available data. Per protocol: participants completing ≥ 2 intervention components.*

## Baseline characteristics

Baseline demographic and clinical characteristics are presented in Table 1. Most adolescents had moderately complex CHD, were born in Sweden, and lived with both parents. No statistically significant differences were observed between the IG and CG at baseline across demographic or clinical variables (all p > 0.05).

## Overall transition readiness

### 1.1 Intention-to-Treat (ITT)

No significant difference in overall transition readiness was observed between groups at baseline (T0; p = 0.383, r = 0.077). At T1, the IG showed a slightly higher mean rank than the CG, but the difference was not statistically significant (p = 0.230). At T2 (age 18.5 years), the IG demonstrated significantly higher transition readiness compared with the CG (p = 0.015, r = 0.227) (Table 2).

**Table 2:** Overall transition readiness scores by group and time point (ITT).

Time point	**Group**	**n**	Median (IQR)	Mean Rank	**U**	p-value	**r**
**T0 (Baseline)**	Intervention	62	5.00 (2.25)	67.94	1895.00	0.383	0.077
	Control	67	5.00 (2.00)	62.28			
**T1**	Intervention	55	6.00 (3.00)	61.28	1414.50	0.230	0.112
	Control	59	6.00 (2.00)	53.97			
**T2**	Intervention	54	7.00 (2.00)	65.78	1227.00	**0.015**	**0.227**
	Control	61	7.00 (3.00)	51.11			

## Full analysis set (FAS)

FAS analyses yielded similar findings. No differences were observed at T0 (p = 0.595) or T1 (p = 0.260). At T2, transition readiness was significantly higher in the IG compared with the CG (p = 0.011, r = 0.24) (Table 3).

**Table 3:** Overall transition readiness scores by group and time point (FAS).

Time point	**Group**	**n**	Median (IQR)	Mean Rank	**U**	p-value	**r**
**T0 (Baseline)**	Intervention	52	5.00 (2.00)	57.70	1445.50	0.595	0.05
	Control	59	5.00 (2.00)	54.50			
**T1**	Intervention	54	6.00 (3.00)	59.72	1338.00	0.260	0.12
	Control	57	6.00 (2.00)	52.47			
**T2**	Intervention	54	7.00 (2.00)	65.56	1185.00	**0.011**	**0.24**
	Control	60	6.50 (2.75)	50.25			

## Transition readiness over time

Mixed-design ANOVA analyses (identical for ITT and FAS datasets) demonstrated a significant main effect of time (F (2212) = 47.19, p < 0.001, η²p = 0.31), indicating that overall transition readiness increased across adolescence in both groups (Fig. 4).

**Fig. 4:**
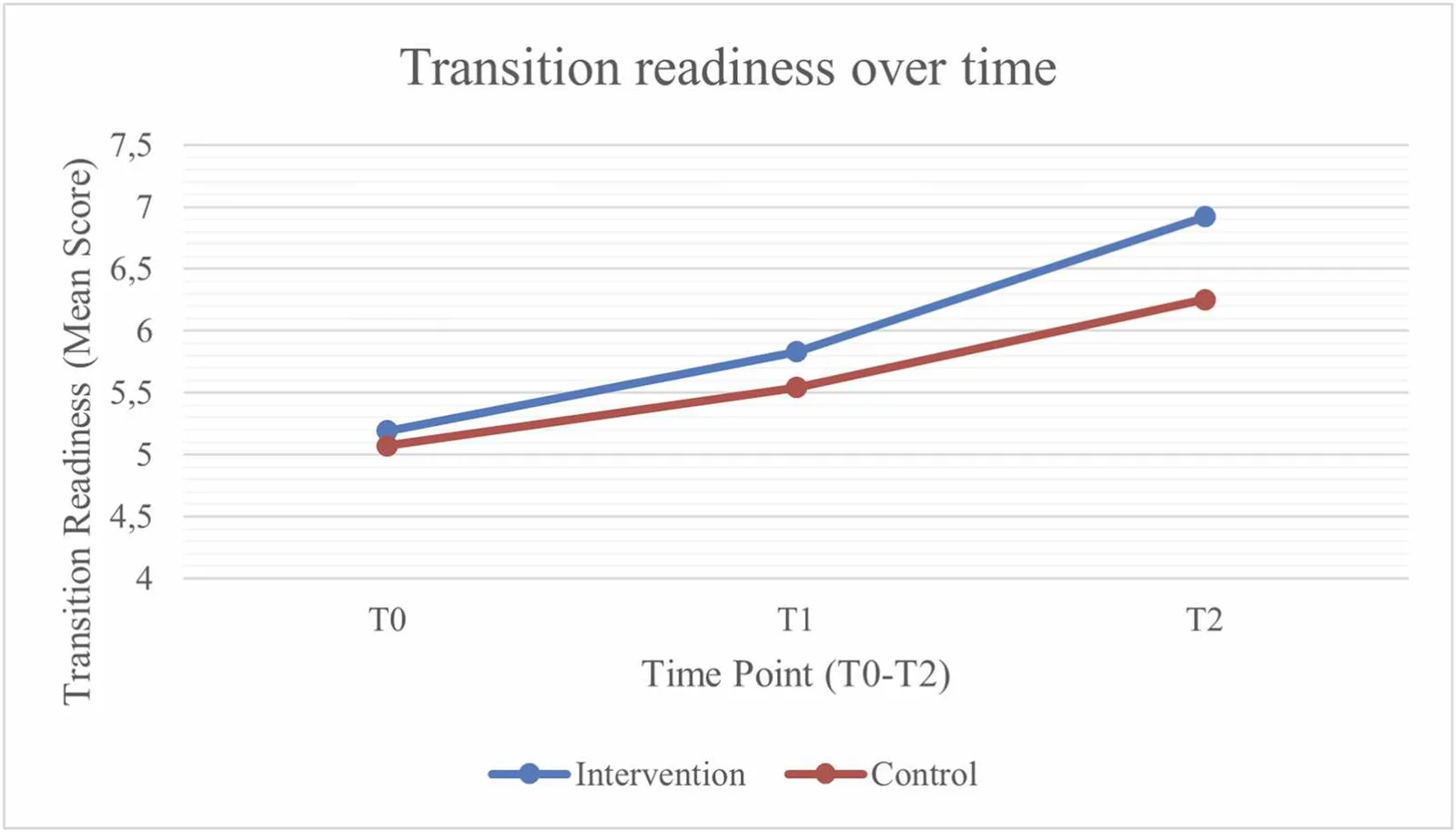
Mean transition readiness scores across T0, T1, and T2 for intervention and control groups. The estimated marginal means increased in the IG from 5.19 at T0–6.92 at T3 (+1.73), while the control group increased from 5.07 to 6.25 (+1.18). Although the increase was slightly greater in the IG, the interaction between time and group was not statistically significant (*p* = 0.177), indicating that the pattern of improvement over time did not differ significantly between the groups (Fig. 4).

No significant main effect of group was observed (p = 0.128, η²p = 0.02), and the group-by-time interaction was not statistically significant (p = 0.177, η²p = 0.02), suggesting similar developmental trajectories in both groups (Table 4).

**Table 4:** Mixed-design ANOVA results for transition readiness.

Source	**F**	**df**_**1**_	**df**_**2**_	**p**	**η**^**2**^_**p**_
**Time**	47.19	2	212	< 0.001	0.31
**Group**	2.35	1	106	0.128	0.02
**Time x Group**	1.75	2	212	0.177	0.02

Estimated marginal means increased from 5.19 to 6.92 (+1.73) in the IG and from 5.07 to 6.25 (+1.18) in the CG. Although the increase was numerically greater in the IG, the interaction effect was not significant.

## Individual RTQ items

At T2, no statistically significant differences were observed between groups for individual RTQ items in either ITT (Table 5) or FAS analyses (Table 6). Effect sizes were small (r = 0.01–0.18), indicating comparable levels of autonomy-related behaviors such as medication management, appointment scheduling, and communication with healthcare providers.

**Table 5:** Group differences in individual RTQ items (ITT).

Variable	IG Mean Rank	CG Mean Rank	U	p	r
**Self-Medication**	27.94	23.06	251.5	0.200	0.18
**Self-Booking Specialist**	38.48	42.53	881.0	0.296	0.12
**Self-Prescription**	32.81	33.21	533.5	0.912	0.01
**Self-Booking Clinic**	47.04	49.96	1222.0	0.522	0.07
**Self-Explanation**	52.30	62.71	1934.5	0.077	0.17
**Self-Visits**	65.90	58.52	1659.0	0.204	0.11
**Self-Communication**	62.47	67.27	2228.5	0.438	0.07
**Self-Phone Communication**	53.14	51.89	1319.0	0.807	0.02

*Effect size calculated with Cohen´s d. Cutoffs were 0.2–0.5 (small effect), 0.5–0.8 (moderate effect) and > 0.8 (large effect).

**Table 6:** Group differences in individual RTQ items (FAS).

Variable	IG Mean Rank	CG Mean Rank	U	p	r
**Self-Medication**	24.83	21.09	211.0	0.299	0.16
**Self-Booking Specialist**	34.74	37.37	582.5	0.453	0.09
**Self-Prescription**	28.81	28.17	382.5	0.853	0.03
**Self-Booking Clinic**	41.05	44.02	819.00	0.475	0.08
**Self-Explanation**	48.01	52.54	1125.5	0.409	0.08
**Self-visits**	56.65	50.79	1242.00	0.277	0.11
**Self-Communication**	54.36	59.33	1450.00	0.389	0.08
**Self-Phone Communication**	47.42	43.58	926.00	0.412	0.09

*Effect size calculated with Cohen´s d. Cutoffs were 0.2–0.5 (small effect), 0.5–0.8 (moderate effect) and > 0.8 (large effect).

## Discussion

This study evaluated the efficacy of the STEPSTONES transition program on adolescents’ transition readiness compared with usual care through an RCT. The main findings were that adolescents in the intervention group demonstrated significantly higher overall transition readiness at age 18.5 years. Although the mixed-design ANOVA did not show a significant group by time interaction effect, the group difference at the final measurement suggests that participation in the program may have contributed to improved readiness prior to transfer. No significant differences were identified at the level of individual RTQ items.

The discrepancy between significant total scores and non-significant item-level findings is noteworthy. The intervention may have produced small improvements across several domains that became detectable only when aggregated into the overall readiness score. Individual items are more susceptible to random variation and measurement error, whereas the total score captures shared variance across domains. Thus, the program’s impact may reflect broader developmental and cognitive shifts rather than discrete behavioral changes.[22], [33]

The observed effect size at T2 was small (r = 0.23), indicating modest practical impact. Nevertheless, even small improvements in readiness may be clinically meaningful. Transition readiness has been associated with increased autonomy, improved medication knowledge, and reduced barriers to adherence.[22], [34] For pediatric clinicians, incremental gains in adolescents’ preparedness may contribute to safer transfer processes and sustained follow-up in adult care.

Previous research demonstrates that structured transition interventions can improve empowerment, knowledge, self-care skills, and continuity of care.[15], [20], [35], [36] Mechanisms identified within the STEPSTONES program include person-centered care coordination, tailored education, and peer support.^32^ Although earlier studies have shown improvements in outcomes relevant to transitional care,^20^ the present findings suggest that effects may be more diffuse, potentially reflecting gradual strengthening of adolescents’ confidence and responsibility rather than immediate changes in task-specific behaviors. Limited statistical power, relatively high baseline scores, and the follow-up duration may also explain the absence of item-level differences.

## Strengths and limitations

As transition readiness was a secondary outcome, the study was not powered specifically for this measure, increasing the risk of type II error. Non-significant findings should therefore be interpreted cautiously. The RTQ was validated in adolescents following kidney transplantation rather than CHD. However, transition-related challenges are largely diagnosis-independent, and adolescents with chronic conditions share developmental and psychosocial needs.^37^ RTQ’s generic structure supports its applicability across chronic illness groups. Finally, the RCT had a response rate of 36% which might have implications for external validity. However, the attrition analysis showed only differences between participants and non-participants in disease-severity, where participants in the RCT had a higher complexity of their CHD compared to non-participants.^10^

## Clinical implications

The findings suggest that structured, person-centered transition programs delivered by trained pediatric nurses may strengthen adolescents’ overall readiness for adult care. More RCTs of transitional care interventions are showing promising results on patient-reported outcomes,[15], [17], [38] and this study adds to this body of knowledge. Pediatric nurses are central in promoting autonomy, self-management, and shared decision-making. Even small improvements in readiness may reduce the risk of lapses in care, which are associated with increased morbidity.^39^ Integrating structured transition processes into routine pediatric cardiology practice may therefore support safer and more equitable transfer. Yet, implementation requires investment in trained personnel encompassing the whole interdisciplinary team, coordination between pediatric and adult services, and protected time for delivering transitional care. Hence, variations in local resources may affect equitable delivery. Ensuring access to structured transition support across regions is therefore an important health system consideration.

## Conclusion

Adolescents with CHD who participated in the STEPSTONES program demonstrated higher overall transition readiness prior to transfer to adult healthcare compared with those receiving only usual care. Although the effect size was small, the findings suggest that a structured, person-centered transition program may strengthen adolescents’ preparedness for adult-oriented services.

Given the risks associated with discontinuity of care during transfer, even incremental improvements in readiness may be clinically meaningful. Transitional support may therefore play an important role in promoting safer, more coordinated, and sustainable transitions for adolescents with CHD. While the findings should be interpreted cautiously, they support further development and evaluation of structured transition programs as a complement to standard pediatric cardiology care.

## Abbreviations

ACHD – Adults with Congenital Heart Disease

CCCG – Contamination Control Group

CG – Control Group

CHD – Congenital Heart Disease

DCO – Data Collection Officer

IG – Intervention Group

STEPSTONES – Swedish Transition Effects Project Supporting Teenagers with chrONic mEdical conditionS

SWEDCON – Swedish Congenital Heart Disease Registry

RCT – Randomized Controlled Trial

RTQ – Readiness for Transition Questionnaire

TC – Transition Coordinator

## CRediT authorship contribution statement

**Hilda Malmesjö:** Writing – review & editing, Writing – original draft, Visualization, Methodology, Formal analysis, Data curation. **Mariela Acuna Mora:** Writing – review & editing, Visualization, Validation, Methodology, Investigation, Formal analysis, Data curation, Conceptualization. **Ewa-Lena Bratt:** Writing – review & editing, Visualization, Validation, Supervision, Software, Resources, Project administration, Methodology, Investigation, Funding acquisition, Formal analysis, Data curation, Conceptualization. **Carina Sparud-Lundin:** Writing – review & editing, Validation, Supervision, Project administration, Methodology, Investigation, Funding acquisition, Conceptualization. **Philip Moons:** Writing – review & editing, Validation, Supervision, Resources, Project administration, Methodology, Investigation, Funding acquisition, Formal analysis, Conceptualization. **Markus Saarijärvi:** Writing – review & editing, Writing – original draft, Validation, Supervision, Project administration, Methodology, Investigation, Formal analysis, Data curation, Conceptualization.

## Ethics declaration

Written informed consent to take part in the study and to publish the article has been obtained from all participants or their legal representatives. The privacy rights of participants have been observed.

This study was performed in compliance with relevant laws, regulatory frameworks and guidelines where the research took place. This study was conducted in accordance with Declaration of Helsinki. This study was approved by the Regional ethical review board in Gothenburg, Sweden. (Approval No. No. 931–15, T-417–16, T-435–17))

The results of this clinical trial and any associated work have been posted in a registry. This clinical trial was registered with number NCT02675361. (Clincal Trials Gov).

## Ethical considerations

The study followed the Declaration of Helsinki^31^ and was approved by the Regional Ethical Review Board in Gothenburg, Sweden (No. 931–15, T-417–16, T-435–17). Written informed consent was obtained from adolescents and parents prior to participation. Participants could withdraw at any time. Although 16-year-olds are legally able to consent under Swedish law, ethical considerations regarding voluntariness and comprehension were carefully addressed. The intervention posed minimal risk, consisting of structured nurse-led support without physical procedures. TCs referred participants to appropriate professionals when needed. Registry data from SWEDCON were used in accordance with opt-out procedures. Confidentiality was ensured through coded data storage and secure handling procedures.^20^

## Declaration of Generative AI and AI-assisted technologies in the writing process

The authors used Chat-GPT and Perplexity for copy and language editing.

## Funding

The work presented in this article was supported by research grants from (a) the 10.13039/501100003793Swedish Heart-Lung Foundation (grant 20150535); (b) Swedish Research Council for Health, Working Life and Welfare-FORTE (grant STYA-2015/0003); (c) the Swedish Children's Heart Association; (d) the 10.13039/501100004359Swedish Research Council (grant 2015–02503). The funding bodies have not participated in study design, collection and analysis of data or writing the manuscript.

## Declaration of Competing Interest

The authors declare that they have no known competing financial interests or personal relationships that could have appeared to influence the work reported in this paper.

## Data Availability

Data will be made available on request.
